# High-Surface-Area-Activated Carbon Derived from Mango Peels and Seeds Wastes via Microwave-Induced ZnCl_2_ Activation for Adsorption of Methylene Blue Dye Molecules: Statistical Optimization and Mechanism

**DOI:** 10.3390/molecules27206947

**Published:** 2022-10-17

**Authors:** Nur Shakinah Razali, Ahmed Saud Abdulhameed, Ali H. Jawad, Zeid A. ALOthman, Tarek A. Yousef, Omar K. Al-Duaij, Norah Salem Alsaiari

**Affiliations:** 1 Faculty of Applied Sciences, Universiti Teknologi MARA, Shah Alam 40450, Malaysia; 2 Department of Medical Instrumentation Engineering, Al-Mansour University College, Baghdad 10068, Iraq; 3 College of Engineering, University of Warith Al-Anbiyaa, Karbala 56001, Iraq; 4 Chemistry Department, College of Science, King Saud University, Riyadh 11451, Saudi Arabia; 5 Department of Chemistry, Science College, Imam Mohammad Ibn Saud Islamic University (IMSIU), P.O. Box 90950, Riyadh 11623, Saudi Arabia; 6 Department of Chemistry, College of Science, Princess Nourah bint Abdulrahman University, P. O. Box 84428, Riyadh 11671, Saudi Arabia

**Keywords:** activated carbon, Mango wastes, ZnCl_2_ activation, adsorption mechanism, methylene blue, Box-Behnken design

## Abstract

In this study, Mango (*Mangifera indica*) seeds (MS) and peels (MP) seeds mixed fruit wastes were employed as a renewable precursor to synthesize high-surface-area-activated carbon (MSMPAC) by using microwave-induced ZnCl_2_ activation. Thus, the applicability of MSMPAC was evaluated towards the removal of cationic dye (methylene blue, MB) from an aqueous environment. The key adsorption factors, namely A: MSMPAC dose (0.02–0.1 g), B: pH (4–10), and C: time (5–15 min), were inspected using the desirability function of the Box-Behnken design (BBD). Thus, the adsorption isotherm data were found to correspond well with the Langmuir model with a maximum adsorption capacity of (232.8 mg/g). Moreover, the adsorption kinetics were consistent with both pseudo-first-order and pseudo-second-order models. The spontaneous and endothermic nature of MB adsorption on the MSMPAC surface could be inferred from the negative ∆G° values and positive value of ∆H°, respectively. Various mechanisms namely electrostatic forces, pore filling, π-π stacking, and H-bonding govern MB adsorption by the MSMPAC. This study demonstrates the utility of MS and MP as renewable precursors to produce high-surface area MSMPAC with a potential application towards the removal of cationic organic dyes such as MB.

## 1. Introduction

Activated carbon (AC) is a common form of carbonaceous material and is widely used in several industrial applications due to its high surface area, abundance of significant functional groups, porous structure, and exceptional adsorption capacity [1]. The AC’s significant adsorption effectiveness and usability make it a vital adsorbent in wastewater treatment methods [2]. Commercial ACs are usually made from pricey and non-renewable resources (e.g., coal, petroleum coke, and peat) [3]. However, the associated financial and environmental problems with AC obtained from the foregoing substances, extensive studies were conducted to seek inexpensive feedstock, i.e., biomass wastes to make AC [4,5]. 

The agricultural biomass materials might reach 70 million tons annually in Malaysia [6]. Mango (*Mangifera indica*) is widely available in Malaysia and produces a sizable amount of mango seeds (MS) and mango peels (MP) every year [7]. Since they slowly and gradually ferment and generate unpleasant aromas, the large volume of MS and MP that must be disposed of is a serious problem in the environment [8]. Thus, producing AC from renewable, low-cost, and feasibly accessible precursors (MS and MP) represents the optimal way of exploiting agricultural waste and getting advantages for the economy and the environment. 

A variety of biomass wastes have recently been used as precursors to produce AC, including pumpkin seed shells [9], corn stalks [10], green alga [11], cottonseed husks [12], *Pisum sativum* pods [13], mangosteen peels [14], and *Halodule uninervis* seagrass [15]. The physicochemical properties of AC, like surface area, high porosity, and adequate oxygen-containing functional groups, are greatly influenced by the preparation conditions of AC, i.e., the type of chemical activator (e.g., H_2_SO_4_, ZnCl_2_, FeCl_3_, and H_3_PO_4_) and precursor character [16]. In fact, ZnCl_2_ contributes to the formation of AC with favorable characteristics such as greater surface area, smaller pore size, and pore volume, as well as increased carbon yield and a large number of reactive groups for absorbing contaminants [17]. Additionally, microwave radiation was the method employed in this study because it has a number of advantageous features, including minimal processing, high final product amount, process control, superior heat transfer, homogenous heating performance, and power density [18]. 

Numerous contaminants, comprising chlorinated substances, organic dyes, heavy metals, surfactants, and inhibitory substances are present in the industrial effluent from the manufacturing sectors, which is the main cause of water pollution [19,20,21]. The residual organic dyes can enter the human body via the food chain and lead to a number of hazardous disorders, including mutation or cancer, skin irritability, permanent blindness, vomiting, hypertension, and gastritis [22]. 

Therefore, before delivering the dye-containing effluent into the environment, it must be treated. For wastewater treatment containing organic dyes, a number of approaches have been developed and effectively used, including photocatalytic degradation [23], ion exchange [24], Fenton-like oxidation [25], adsorption [26], and membrane filtering [27]. Due to its excellent performance, cost-effectiveness, capacity to recover and repurpose adsorbents, diverse applicability, and simplicity of usage, adsorption has been recognized as a significant way to remove those pollutants [26]. Response surface methodology (RSM) combined with Box-Behnken design (BBD) was introduced to reduce the laborious adsorption testing approach and improve an operator’s ability to understand potential interactions between various factors [28]. 

The aforementioned data inspires the current work for the production of activated carbon (thereafter, defined as MSMPAC) from MS and MP biomass wastes using microwave-assisted ZnCl_2_ activation. The prepared MSMPAC was employed to remove MB dye from the simulated wastewater. Key adsorption parameters (MSMPAC dose, pH, and time) were optimized using the Box-Behnken design’s numerical desirability function (BBD). The adsorption ability of the created MSMPAC and the MB adsorption mechanism was deduced using adsorption isotherm, kinetic, and thermodynamic analyses of the MB adsorption.

## 2. Materials and Methods

### 2.1. Materials

Mango seeds (MS) and mango peels (MP) used as precursors to produce AC were collected from juice shops in Shah Alam, Malaysia. The exact amount of 1 g of MB powder was dissolved in 1 L of deionized water to prepare a stock solution (1000 mg/L) of the MB (MW: 319.86 g/mol; R&M Chemicals; λ_max_: 661 nm). Thus, R&M Chemicals, Malaysia supplied the chemicals and reagents such as sodium chloride (NaCl), zinc chloride (ZnCl_2_), sodium hydroxide (NaOH), and hydrochloric acid (HCl).

### 2.2. MSMPAC Synthesis

The samples (MS) and (MP) were washed with boiled distilled water before being subjected to the drying process inside an oven for 24 h at 100 °C. Then, the samples (MS) and (MP) were subsequently ground into a powder with a consistent particle size (2 mm) before being mixed with a fixed ratio of 50% MS + 50 % MP to produce the final form of (MSMP) powder. After that, the chemical activation process was carried out by mixing 1 g of MSMP and 2 g of ZnCl_2_ to achieve a 1:2 mixing ratio (pre-determined as the best ratio). Then, the mixture of the MSMP + ZnCl_2_ was placed inside an oven (100 °C) for 48 h before being subjected to thermal activation. In the activation process, a fabricated microwave oven (SAMSUNG ME711K, 20 L, Seoul, South Korea) was used to activate MSMP + ZnCl_2_ system at a fixed microwave radiation power (800 W) and radiation time (20 min) under pure N2 (99.99) ,condition with a fixed flow rate of 100 mL/min. Thus, the produced MSMPAC was then rinsed with distilled water until the pH was neutral. The moisture content was then removed by heating MSMPAC for 24 h at 100 °C. Finally, a powder with a 250 µm particle size of MSMPAC was produced by grinding and sieving the MSMPAC to the desired particle size. 

### 2.3. Characterization

The porosity and surface analyzer (Micromeritics, ASAP 2060, Norcross, GA, USA), characterized the MSMPAC’s specific surface area and porosity. The chemical nature of the MSMP and MSMPAC adsorbent prior to and during MB uptake was explored using Fourier transform infrared (FTIR) (Perkin-Elmer, Spectrum RX I, Waltham, MA, USA). The pH at which the MSMPAC surface has a net neutral charge was obtained by determining the “point of zero charge (pH_pzc_)” [29]. With the use of a Zeiss SEM (Model Supra 40 VP), the topology of the MSMPAC and MSMPAC-MB surfaces was examined. X-ray diffraction (XRD) analysis using the PANalytical X’Pert PRO model was employed to identify the crystallinity of the MSMPAC. 

### 2.4. Experimental Design

Response surface methodology (RSM) is a statistical method for examining how removal process factors interact and determining the best model parameters that maximize the removal efficiency (%) of MB dye. Using the statistics tool Design Expert 13 (version 13, State Ease, Minneapolis, MN, USA), the investigative strategy and statistical analysis were conducted. Table 1 displays the grades and names (with their codes) of the assessed variables. 

The model’s quadratic function (Equation (1)) of the inputs and output is denoted in Equation (1).

(1) $$Y=\beta_{0}+\sum \beta_{i}X_{i}+\sum \beta_{ii}X_{i}^{2}+\sum \sum \beta_{ij}X_{i}^{\;}X_{j}$$

where *Y* is MB removal (predicted); *β*_0_ = constant; *X_i_* and *X_j_* denote the evaluated parameters; *β_i_* signifies linear impact coefficient; *β_ii_* symbolizes quadratic impact coefficient; *β_ij_* indicates interaction impact coefficient. Table 2 displays experimental matrix of BBD and corresponding quadratic model response (MB removal). 

The MB dye removal tests started with the addition of various adsorbent quantities to 100 mL of modeled contaminants containing 100 mg/L of MB, which were then shaken in a water bath for a fixed period. After that, liquids were processed through a 0.45 μm syringe to remove the adsorbent. The remnant MB concentration in the proposed pollutant sample was measured using a UV-Vis spectrometer (HACH DR 2800) at a maximum wavelength of 661 nm. The removal rate (R %) of MB dye was computed using Equation (2).

(2) $$R\;\%=\frac{\left(C_{o}-C_{e}\right)}{C_{o}\left(2\right)}\times 100$$

where the concentrations of MB (mg/L) throughout their initial and equilibrium phases are represented as $C_{o}$ and $C_{e}$, respectively.

### 2.5. Adsorption Study of MB on MSMPAC

Quantifying the amount of MB adsorbed onto the MSMPAC based on the numerical desirability function, batch adsorption investigations have been carried out. The desirability function data stated that the best conditions for the maximum removal of MB (98.1%) were MSMPAC dose (0.09 g), pH (8.7), and duration (14.8) min. As a result, adsorption experiments were fulfilled under these ideal circumstances using different MB beginning concentrations (20–200 mg/L). The batch adsorption investigations for MB dye were undertaken using the same methods described in the section before this one (2.4). Hence, the adsorption capacity (*q_e_*, mg/g) of the MSMPAC was estimated by using Equation (3).

(3) $$q_{e}=\frac{\left(C_{o}-C_{e}\right)V}{W}$$

where terms *V* (L) and *W* (g) refer to the MSMPAC’s quantity and volume of solution, respectively.

## 3. Results and Discussion

### 3.1. Characterization of MSMPAC

The data of MSMPAC’s pore structure and surface area are provided in Table 3. According to measurements, MSMPAC has a total pore volume of 0.614 cm^3^/g and a BET surface area of 1151.6 m^2^/g. According to IUPAC [30], the MSMPAC possesses mesopores (pores ranging 2–50 nm) owing to its mean pore diameter value of 3.05 nm. 

**Figure 1:**
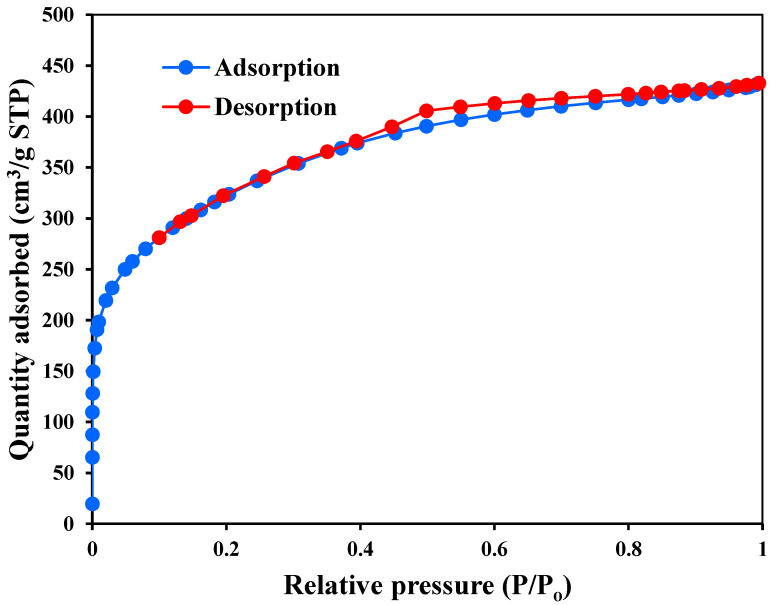
N_2_ adsorption-desorption isotherms and pore size distribution of MSMPAC.

The N_2_ adsorption-desorption isotherm curve of MSMPAC is displayed in Figure 1. As per the IUPAC classification, Figure 1 depicts a Type I isotherm with a Type 4 hysteresis loop [30]. Due to the micropore filling effect, the adsorption trend of MSMPAC exhibits a sharp increase in the low relative pressure range while, at high pressure, the trend of the plot exhibits multilayer sorption and capillary condensation, implying the existence of mixed micro- and mesopores within the [31]. The reversible adsorption of molecules in holes with sizes that are near the diameter of nitrogen molecules may be the cause of the minor hysteresis loop [10]. The significant surface area (1151.6 m^2^/g) covered by micropores is further evidence that Type I isotherm is often linked to microporosity. This demonstrates that MSMPAC is constituted of a combination of micropores and mesopores. An additional indication of the presence of narrow slit-like mesopores in the structure of MSMPAC is the fact that capillary condensation took place at a relative pressure (P/P_o_) of above 0.7. Additionally, BJH (Barrett, Joyner, Halenda) pore size distributions (inserted curve in Figure 1) might be employed to conclusively prove that MSMPSAC contains micro and mesopores. The majority of the holes produced in the MSMPAC formed with the ZnCl_2_ agent were tiny, which may be explained by the small particle size of ZnCl_2_ and its hydrates [32]. As per the aforementioned information, the produced MSMPAC has a high degree of porosity, a huge pore volume, and a massive specific surface area. Thus, it offers a sizable number of active sites that can effectively interact with MB dye. 

**Figure 2:**
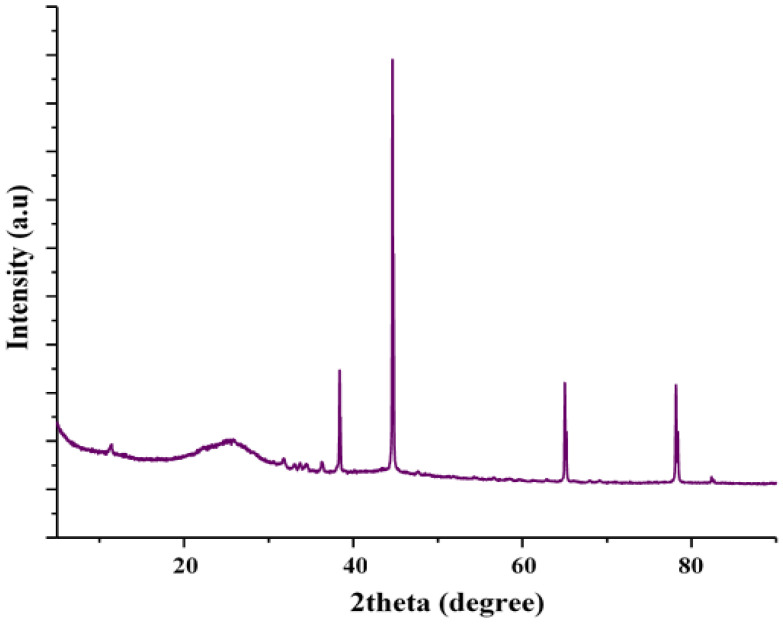
XRD pattern of MSMPAC.

The crystallinity of MSMPAC was characterized using the XRD technique. Figure 2 expresses the XRD pattern of MSMPAC. The amorphous phase of the MSMPAC structure is shown by the broad (002) diffraction peak that existed at 2θ = 24°, whereas the strong (101) diffraction peak that formed at 2θ = 44° may be ascribed to the MSMPAC’s graphite structure [33]. Furthermore, it has been found that MSMPAC exhibits notable peaks at 2θ = 31.9°, 34.5°, 36.3°, 63.14°, 66.5°, and 77.2°, which are linked to an activator’s (ZnCl_2_) role in the process of MSMPAC formation and correspond to ZnO [34]. 

**Figure 3:**
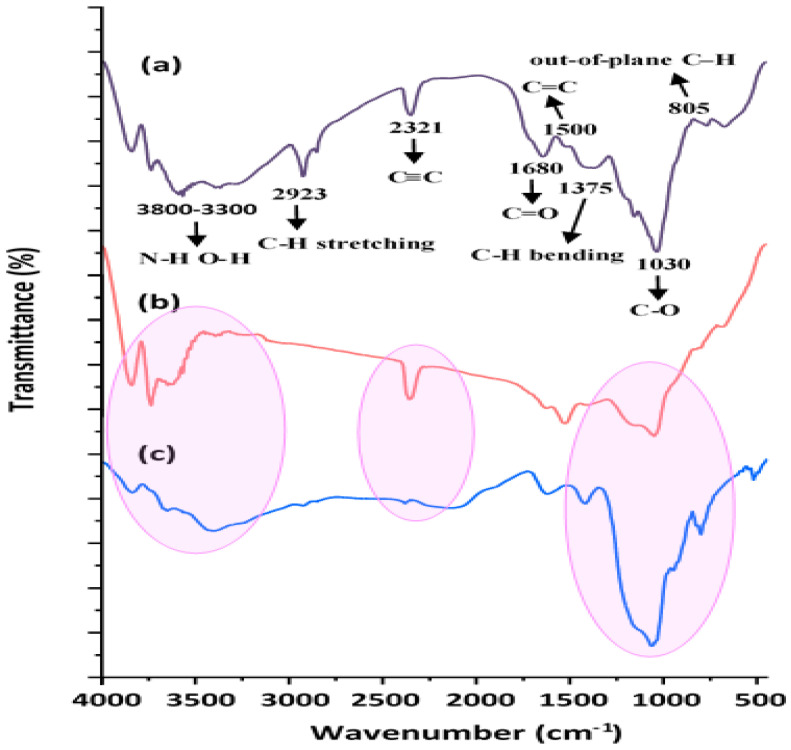
FTIR spectra of (**a**) MSMP, (**b**) MSMPAC, and (**c**) MSMPAC-MB.

FTIR spectroscopy was used to identify the surface functional groups of the pristine (MSMP), MSMPAC, and MSMPAC-MB samples. The FTIR spectra of unprocessed MSMP, MSMPAC, and MSMPAC-MB are shown in Figure 3a–c. The O-H stretching vibrations of cellulose, pectin, hemicellulose, lignin, and absorbed water as well as N-H stretching vibrations of amine groups are responsible for the broad peak (Figure 3a) in the range from 3800 to 3300 cm^−1^ [33]. The asymmetric C-H stretching and C-H bending of the -CH_2_- and -CH_3_ groups, respectively, can be attributed to the weak peak at about 2923 cm^−1^ and the medium band at 1375 cm^−1^ [35]. The stretching vibration of C=O and C=C in bioactive molecules may be responsible for the peaks at 1680 cm^−1^ and 1500 cm^−1^. The peak at 2321 cm^−1^ is assigned to alkyne group C≡C. The sharp peak at 1030 cm^−1^ and the weak band at 805 cm^−1^ are attributed to C-O stretching vibration and out-of-plane C-H derivatives, respectively [7]. The FTIR spectra of MSMPAC (Figure 3b) exhibited a slight difference in the intensity of several peaks compared to MSMP especially peaks at 1500 cm^−1^ (C=C stretch was boosted) and 1030 cm^−1^ (C-O stretching was minimized), implying that chemical bonds were destroyed across MSMPAC’s production. The FTIR spectra of MSMPAC after MB adsorption (Figure 3c) are essentially the same as those of MSMPAC but with a marked shift in numerous bands. The bands’ shift indicates that the MSMPAC’s main functional groups heavily participated in MB adsorption. 

**Figure 4:**
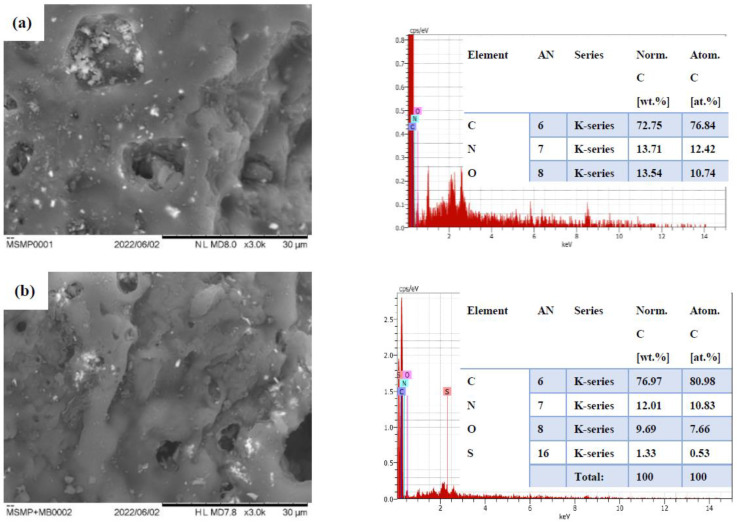
SEM images and EDX spectra of (**a**) MSMPAC and (**b**) MSMPAC-MB at a magnification of 3.0 k.

To determine the morphological characteristics and chemical composition of MSMPAC and MSMPAC-MB, SEM-EDX analysis was used. The SEM image and EDX spectra of MSMPAC are shown in Figure 4a, whereas those of MSMPAC-MB are shown in Figure 4b. The MSMPAC surface (Figure 4a) has a surface that is mostly smooth with few cracks and cavities. A ess porous structure was generated on the MSMPAC-MB surface than on the MSMPAC sample, signifying that the MSMPAC surface was successfully covered by MB molecules. The S atom (sourced from the MB dye) was observed in MSMPAC-MB, whereas the C, N, and O atoms in MSMPAC and MSMPAC-MB were affirmed by EDX data.

### 3.2. Model Fitting

Analysis of variance (ANOVA) was conducted to estimate the validity of the model and assess the strength of explored variables and their interactions. Table 4 lists the ANOVA findings of the derived second-order mathematical model. The results of the ANOVA (Table 4) demonstrate that the proposed model has a statistically meaningful (F-value of 31.0). The *R*^2^ value for the BBD model was calculated to be 0.97. The strong link between MB removal findings practically acquired and those estimated by the proposed quadratic model is supported by this finding. The non-significant *p*-values (0.4143) of the Lack of Fit provided evidence for the suitability of the developed model [36]. Commonly, variables that influence MB adsorption rate and seem to have *p*-values *p* < 0.05 are perceived to be statistically meaningless. Therefore, the developed model’s codes A, B, C, AB, AC, and A^2^ were assessed to be statistically meaningful. The second polynomial model provides the three tested variables and the expected MB removal after fitting is shown in Equation (4).

(4) $$\mathrm{MB}\;\mathrm{removal}\;\left(\%\right)=+76.80+10.41A+2.54B+4.03C+2.52\mathrm{AB}+2.65\mathrm{AC}+4.66A^{2}$$

**Figure 5:**
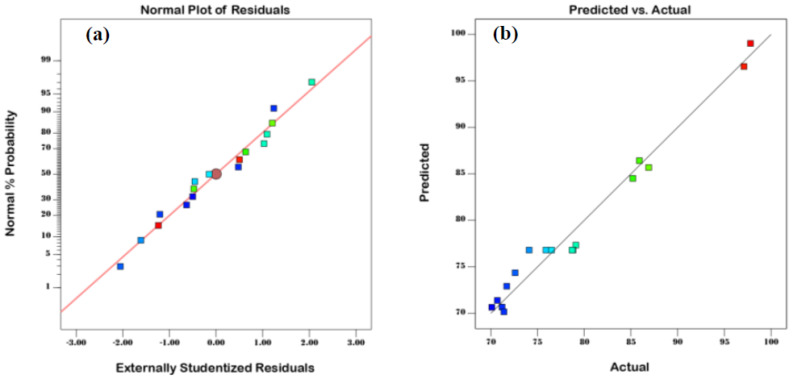
Plots illustrating (**a**) the normal probability of residuals and (**b**) the association between expected and real MB removal levels.

It is crucial to conduct an additional analysis of the established model by investigating the normal probability plot (Figure 5a) to make sure the residuals are normally distributed [37]. Both the model’s design and the results of the ANOVA are fully satisfactory, as shown by the normal distribution of values in Figure 5a around the 45° straight line. Additionally, as seen in Figure 5b, the points representing the real values and predicted values are uniformly spaced on both sides of the line, indicating that the calculated values are consistent with the actual values.

### 3.3. Effects of interactive variables

**Figure 6:**
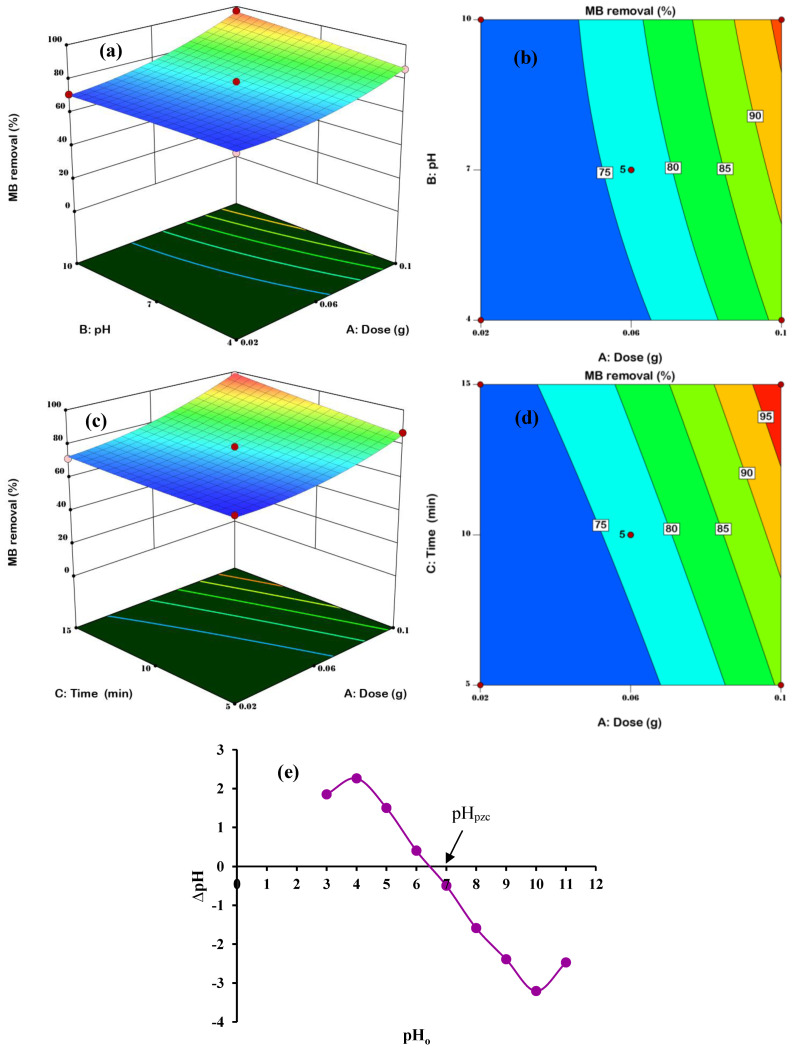
3D response surfaces and 2D contour plots of AB ((**a**): 3D; (**b**): 2D) and AC ((**c**): 3D; (**d**): 2D) interactions; while (**e**) pHpzc of MSMPAC.

3D response surfaces and 2D contour plots were constructed to discover the important interactions between assessed components and to establish their essential properties in the MB removal process. The interaction of MSMPAC with pH strongly impacted the removal process of MB dye as shown in Figure 6 (a: 3D and b: 2D), while the time (10 min) was held constant. The removal of MB was not improved with the change in pH value from 4 to 10, as seen in Figure 6a,b. As shown in Figure 6e, the pH_pzc_ of the MSMPAC was 6.4, further demonstrating that the MSMPAC surface may acquire a positive charge at low pH of 4, i.e., less than pH_pzc_. In contrast, at a solution pH of 10, MSMPAC’s surface charge turns to the negative, demonstrating the MSMPAC’s capacity for cationic dye adsorption. According to the pK_a_ value of MB (pK_a_ = 3.8), the cationic MB molecules were more abundant in environments with pH levels greater than pH_pzc_ [38]. As a result, as shown in Equation (5), stronger electrostatic interactions take place between the cationic MB dye and negatively charged functional groups of MSMPAC.

(5) $$\mathrm{MSMPAC}-O^{-}+{\mathrm{MB}}^{+}⟷\;\mathrm{MSMPAC}-O^{-}\ldots {}^{+}\mathrm{MB}$$

The interaction of MSMPAC with time strongly impacted the removal process of MB dye as shown in Figure 6 (c: 3D and d: 2D), while the pH (7) was held constant. The magnitude of MB removal was slightly increased with higher MSMPAC doses (0.02 g to 0.1 g) according to Figure 6c,d, which may be ascribed to greater numbers of active adsorption sites and larger surface area. Figure 6c,d signifies that the MSMPAC functioned better in removing MB at a higher time (15 min). This is due to the MB molecules taking sufficient time to adsorb on the adsorbent surface and migrate into the MSMPAC mesopores and achieve equilibrium. 

### 3.4. Optimization by the Desirability Functions

**Figure 7:**
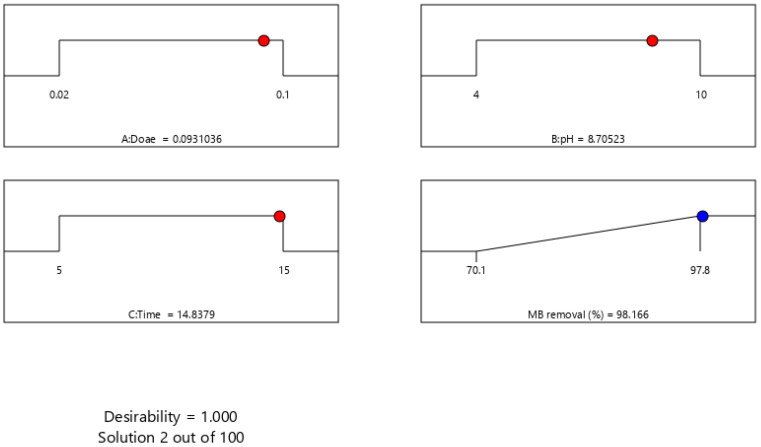
Desirability ramps for optimizing key adsorption factors of MB dye removal (%) using MSMPAC.

Derringer and Suich developed a multi-response optimization method in 1980 called the desirability function, sometimes known as Derringer’s desirability function [39]. Individual desirability (di) values between 0 and 1 are first created for each individual response in this method. A desirability value of 1 generally means that the targeted level of response was attained, whereas a desirability value of 0 means that the desired level of reaction was surpassed [3]. The following Equation (6) is used to determine the total desirability function.

(6) $$D=\left(d_{1}\times d_{2}\times \ldots \times d_{n}\right)\frac{1}{n}=\left(\Pi_{i=1}^{n}d_{1}\right)\frac{1}{n}$$

where D denotes the overall desirability, n represents the count of replies, and di indicates the desirability of each response. The objective is to determine the spot at which desirability has the highest value. Accordingly, a desirability function is a known method for pointing out how to synchronously improve variables such as A: MSMPAC dose, B: pH, and C: time, which achieves the desired efficiency for the response (MB removal) and the detailed information was mentioned in [39]. As per the desirability function, the MSMPAC dosage (0.09 g), pH (8.7), and time (14.8 min) were the factors that successfully removed MB. As shown in Figure 7, under these operating circumstances, the MB decolorization (%) was 98.1 and the desirability value was 1. Duplicate validation experiments with optimized components were used to validate the reliability of this estimation. Overall, the results obtained from empirical observations were in good agreement with the information acquired by numerical optimization. These results illustrate that the BBD model coupled with the desirability function may be effectively used to enhance the MB adsorption by MSMPAC. Consequently, the following investigations employed the optimal input values for MB adsorption. 

### 3.5. Adsorption Study

**Figure 8:**
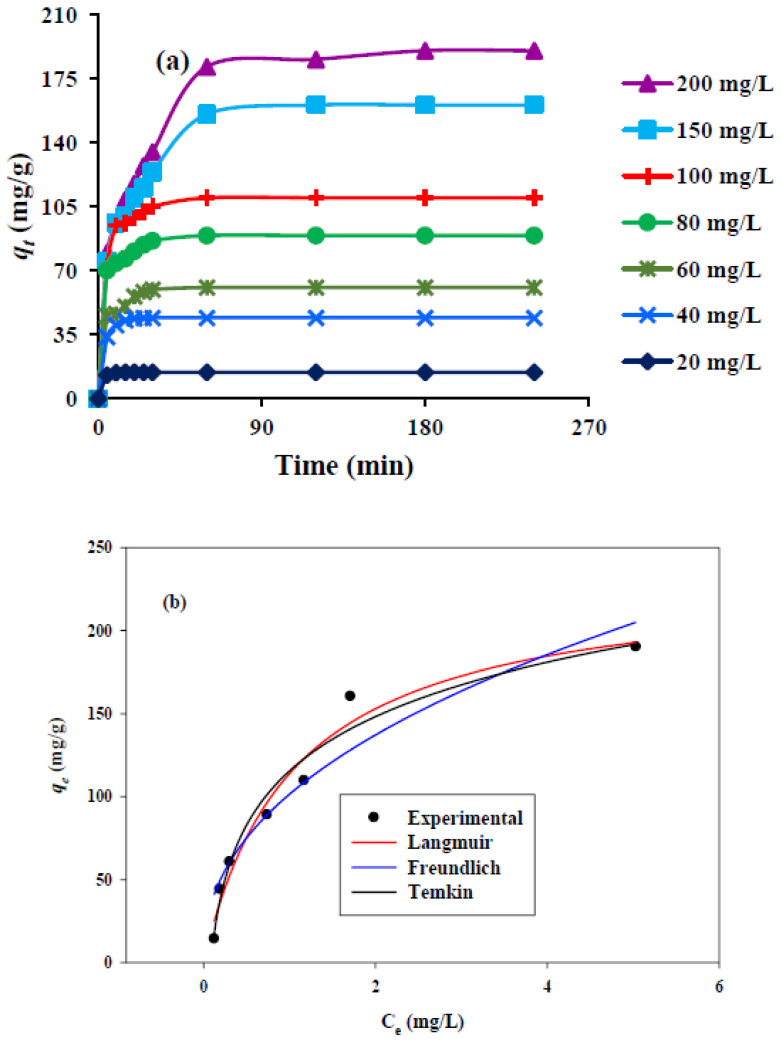
(**a**) Contact-time effect on MB adsorption at various concentrations; (**b**) MB adsorption isotherms (dose = 0.09 g, solution pH = 8.7, temperature = 25 °C, stirring speed = 90 rpm, and volume of solution = 100 mL).

The adsorption rate of the MSMPAC is directly affected by varying the initial MB concentration (20, 40, 60, 80, 100, 150, 200 mg/L), as shown in Figure 8a. The pH of the solution (8.7) and MSMPAC dosage (0.09 g) were kept constant throughout this study. The adsorption capacity of MSMPAC was significantly elevated (14.4 to 190.2 mg/g) because of raising the initial MB concentration from 20 to 200 mg/L. The influence is caused by the MB’s movement to the active adsorption sites and the MB’s increased diffusion within the internal pores of the MSMPAC, which is caused by the higher concentration gradient [37]. 

### 3.6. Adsorption Kinetics

To establish the adsorption mechanism of MB dye on the MSMPAC, the pseudo-first-order [40] and pseudo-second-order [41] models of adsorption kinetics were used. Thus, the PFO and PSO kinetic models (See Table 5) were employed to simulate empirical kinetic data.

Furthermore, Table 6 lists the computed outcomes from the kinetic models. The PSO models obtained *R*^2^ values are marginally closer to those of the experimental data. It was also discovered that the predicted MB dye adsorption capabilities computed using the PSO models are consistent with the experimental values. This implies that the PSO models may adequately explain the process of MB adsorption onto the MSMPAC, showing that chemisorption may be the main step that controls the adsorption rate [42]. 

### 3.7. Adsorption Isotherms

The adsorption isotherm offers significance and helpful familiarity with how the dye molecules are allocated between the MSMPAC and the liquid solution in the equilibrium state and reveals maximizing the adsorption capability of the MSMPAC [43]. In the current work, Langmuir, Freundlich, and Temkin isotherms were assessed. The Freundlich [44], Temkin [45], and Langmuir [46] models were used to designate experimental data. Table 5 summarizes the mathematical formula for each of these model types. Table 7 lists the computed outcomes from the isotherm models, and Figure 8b provides the isotherm curves for the adsorption of MB. Values of 1/n can be used to determine the type of isotherm, such as whether it is irreversible (1/n = 0), advantageous (0 > 1/n), or unfavorable (1/n > 1). In the Freundlich isotherm, the value of 1/n for MB was 0.43, showing an ideal adsorption performance [47]. The experimental data were found to be well-fitted with the Langmuir model based on *R*^2^ values (Table 7). This finding implies that MB adsorption on MSPSAC occurs as a monolayer and at homogenous sites [47].

MSMPAC’s q_max_ for MB was identified to be 176.6 mg/g. The comparison of MSMPAC’s *q*_max_ with other ACs described in previous efforts toward MB is summarized in Table 8. According to the findings in Table 8, MSMPAC is a promising material for the removal of MB from aqueous environments with desirable adsorption capacity over other activated carbon-based adsorbents.

### 3.8. Thermodynamic Characteristics

The removal capability of the prepared MSMPAC is highly impacted by temperature changes; hence, information regarding the nature of the MB adsorption mode is necessary. For this purpose, the obtained adsorption data at varying temperatures (298.15–328.15 K) were employed for computing the thermodynamic characteristics namely, Gibbs free energy (∆G°), entropy (∆S°), and enthalpy (∆H°). The following Equations (7)–(9) were used to find the values of these parameters [53].

(7) $$\Delta G{}^{\circ}=-RT\;lnK_{d}$$

(8) $$k_{d}=\frac{q_{e}}{C_{e}}$$

(9) $$lnk_{d}=\frac{\Delta S{}^{\circ}}{R}-\frac{\Delta H{}^{\circ}}{RT}$$

The intercept and slope of the associated plot of ln *k_d_* against 1/T were used to calculate, respectively, ∆S° and ∆H°. Table 9 outlines the estimated values of thermodynamics. The spontaneous and plausible nature of MB adsorption on the MSMPAC surface could be inferred from the negative ∆G° values [54]. The positive value of ∆H° makes it abundantly evident that the MB uptake phenomenon is endothermic [54]. The growing irregularity at the adsorbent/solution system interface throughout the MB adsorption route is shown by the positive sign of the ∆S°. Additionally, when the temperature rose from 298.15 K to 308.15 K, the adsorption efficiency improved from 92.33 mg/g to 102.02 mg/g. This rise in adsorption capacity was initially rapid, reaching 308.15 K, before slowing down to around 318.15 K. As the temperature rose, the bonds between the pores weakened, resulting in the pores expanding and the appearance of additional active sites. Additionally, heating caused an increase in the rate of MB diffusion into the pore [55]. 

### 3.9. Adsorption Mechanism of MB Dye

**Figure 9:**
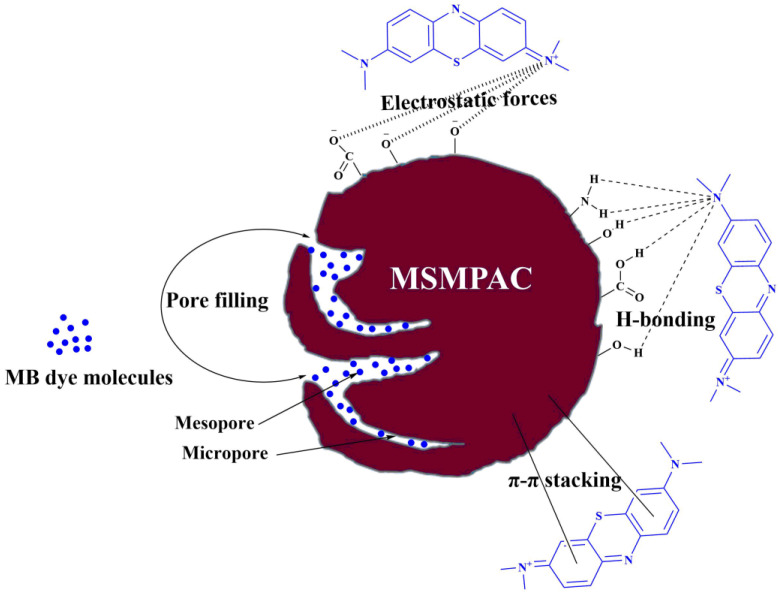
Schematic representation of potential interactions between MSMPAC and MB, including electrostatic forces, pore filling, hydrogen bonding, and π-π stacking.

The adsorption of MB on MSMPAC could be accomplished by various interactions like pore-filling, electrostatic attraction, π–π interaction, and hydrogen bonding. In Figure 9, a schematic illustration of the potential MB adsorption process onto MSMPAC is shown. As per the adsorption tests, the removal of the MB dye molecules occurred in the basic medium (pH greater than the pH_pzc_); thus, in this environment, the surface of MSMPAC will gain negative charges, while MB molecules will bear a positive charge, causing the significant interaction called electrostatic forces, which is considered crucial in MB adsorption. Additionally, MB molecules can be entered into the MSMPAC structure by its micropores and mesopores, and thus, they will subsequently adsorb onto the active sites. As a result, MB adsorption by the pore-filling route is greatly aided by the many mesopores seen in the MSMPAC structure. In order to establish hydrogen bonds, the nitrogen atoms of the MB molecules interact with the hydrogen atoms of the hydroxyl groups presented on MSMPAC’s surface [22]. As a result of interactions between MSMPAC’s hexagonal skeleton and MB’s aromatic backbone, π-π stacking creates, which constitutes a further parameter in increasing the effectiveness of the MB adsorption [56]. 

## 4. Conclusions

Fruit biomass wastes such as MS and MP were efficiently used to generate MSMPAC by microwave induced ZnCl_2_ activation at 800 W for 20 min with an impregnation ratio of 2 g MSMP and 1 g ZnCl_2_. The desirability function data stated that the best conditions for maximum removal of MB (98.1%) were MSMPAC dose (0.09 g), pH (8.7), and duration (14.8) min. The monolayer adsorption process was validated by matching the acquired equilibrium data to the Langmuir model, and the greatest adsorption capacity of 232.8 mg/g was achieved. The spontaneous and endothermic nature of MB adsorption on the MSMPAC surface could be inferred from the negative ∆G° values and positive value of ∆H°, respectively. Various mechanisms namely electrostatic forces, pore filling, π-π stacking, and H-bonding govern MB adsorption by the MSMPAC. This study demonstrates the utility of MS and MP as a renewable precursor for the effective and reliable creation of high surface area MSMPAC and its application in the removal of pollutants from polluted water. 

## Figures and Tables

**Table 1:** Codes and actual variables and their levels in BBD.

Codes	Variables	Level 1 (−1)	Level 2 (0)	Level 3 (+1)
A	MSMPAC dose (g)	0.02	0.06	0.1
B	pH	4	7	10
C	Time (min)	5	10	15

**Table 2:** Experimental matrix of BBD and corresponding quadratic model response (MB removal).

Run	A:Dose (g)	B:pH	C:Time (min)	MB Removal (%)
1	0.02	4	10	70.1
2	0.1	4	10	85.9
3	0.02	10	10	71.2
4	0.1	10	10	97.1
5	0.02	7	5	71.4
6	0.1	7	5	86.9
7	0.02	7	15	71.7
8	0.1	7	15	97.8
9	0.06	4	5	70.7
10	0.06	10	5	72.6
11	0.06	4	15	79.1
12	0.06	10	15	85.2
13	0.06	7	10	74.1
14	0.06	7	10	75.9
15	0.06	7	10	78.8
16	0.06	7	10	76.5
17	0.06	7	10	78.7

**Table 3:** Surface properties of MSMPAC.

Parameter(s)	Value
BET surface area (m^2^/g)	1151.6
Langmuir surface area (m^2^/g)	1585.4
Total volume in pores ( cm^3^/g)	0.614
Mean pore diameter (nm)	3.05

**Table 4:** Analysis of variance (ANOVA) for MB removal (%).

Source	Sum of Squares	df	Mean Square	F-Value	*p*-Value	Remarks
Model	1200.32	9	133.37	31.00	<0.0001	Significant
A-Dose	867.36	1	867.36	201.59	<0.0001	Significant
B-pH	51.51	1	51.51	11.97	0.0105	Significant
C-Time	129.61	1	129.61	30.12	0.0009	Significant
AB	25.50	1	25.50	5.93	0.0451	Significant
AC	28.09	1	28.09	6.53	0.0378	Significant
BC	4.41	1	4.41	1.02	0.3450	Not significant
A²	91.53	1	91.53	21.27	0.0024	Significant
B²	0.6322	1	0.6322	0.1469	0.7128	Not significant
C²	1.00	1	1.00	0.2326	0.6443	Not significant
Residual	30.12	7	4.30			
Lack of Fit	14.32	3	4.77	1.21	0.4143	Not significant
Pure Error	15.80	4	3.95			
Cor Total	1230.44	16				

**Table 5:** Adsorption kinetics and isotherms nonlinear models.

Models	Formula	Descriptions
Pseudo-first-order (PFO)	$q_{t}=q_{e}\left(1-e^{-k_{1}t}\right)$	$k_{1}$: pseudo-first-order rate constant (1/min)
Pseudo-second-order (PSO)	$q_{t}=\frac{q_{e}^{2}k_{2}t}{1+q_{e}K_{2}t}$	$k_{2}$: pseudo-second-order rate constant (g/mg min)
Langmuir	$q_{e}=\frac{q_{m}K_{L}C_{e}}{1+K_{L}C_{e}}$	$q_{m}$: monolayer capacity (mg/g) *K_L_* : Langmuir constant (L/mg)
Freundlich	qe=KFCe1n	*K_F_* : Freundlich constant (mg/g) (L/mg)^1/n^ *n*: adsorption intensity
Temkin	$q_{e}=\frac{RT}{b_{T}}ln\left(K_{T}C_{e}\right)$	$K_{T}$: Temkin constant (L/mg) $b_{T}$: adsorption intensity(J/mol)

**Table 6:** PFO and PSO kinetic parameters for MB adsorption on MSMPAC.

Concentration (mg/L)	*q*_e exp_ (mg/g)	PFO			PSO		
		*q*_e cal_ (mg/g)	*k*_1_ (1/min)	*R* ^2^	*q*_e cal_ (mg/g)	*k*_2_ × 10^−2^ (g/mg min)	*R* ^2^
20	14.4	14.4	0.44	1	14.6	13.0	0.99
40	44.2	44.1	0.27	0.99	45.6	1.11	0.99
60	60.7	58.7	0.22	0.96	61.9	0.69	0.99
80	89.1	85.4	0.29	0.97	89.6	0.64	0.99
100	109.7	106.2	0.21	0.98	112.2	0.36	0.99
150	160.5	154.9	0.07	0.93	166.9	0.07	0.98
200	190.2	184.7	0.05	0.94	200.8	0.04	0.97

**Table 7:** The parameters of isotherm models for MB dye adsorption on MSMPAC.

Adsorption isotherm	Parameter	Value
Langmuir	*q_max_* (mg/g)	232.8
	*K_a_* (L/mg)	0.96
	*R* ^2^	0.97
Freundlich	*K_f_* (mg/g) (L/mg)^1/n^	101.4
	*n*	2.29
	*R* ^2^	0.92
Temkin	*K_T_* (L/mg)	2.44
	*b_T_* (J/mol)	52.5
	*R* ^2^	0.96

**Table 8:** Comparison of the adsorption capacity of MSMPAC towards MB dyes with different ACs.

Adsorbents	*q_max_* (mg/g)	References
MSMPAC	232.8	This study
Activated carbon from green algae *Ulva lactuca*	344.83	[48]
Activated carbon from palm oil waste	188	[49]
Jengkol peel-based activated carbon	170.9	[50]
Activated carbon from *Moringa oleifera* leaf	136.99	[51]
Activated carbon from *Elaeagnus angustifolia* seeds	72	[52]

**Table 9:** Thermodynamic parameters for MB dye adsorption onto MSMPAC.

T (K)	*q*_e_ (mg/g)	$K_{d}$	∆G° (kJ/mol)	∆H° (kJ/mol)	∆S° (kJ/mol·K)
298.15	92.3	4.06	−3.48	18.2	0.074
308.15	102.0	7.92	−5.30		
318.15	103.7	8.23	−5.58		
328.15	103.8	8.34	−5.79		

## Data Availability

The datasets used and/or analyzed during the current study are available from the corresponding author on reasonable request.

## Sample Availability

Samples of the compounds activated carbon derived from mango peels and seeds wastes are available from the authors.

## References

[1] Wang D., Wang Z., Zheng X., Tian M. (2020). Activated carbon fiber derived from the seed hair fibers of *Metaplexis japonica*: Novel efficient adsorbent for methylene blue. Ind. Crops Prod..

[2] Yang R., Zhou J., Wu L., Ping S. (2022). Fabrication of developed porous carbon derived from bluecoke powder by microwave-assisted KOH activation for simulative organic wastewater treatment. Diam. Relat. Mater..

[3] Jawad A.H., Abdulhameed A.S., Hanafiah M., ALOthman Z.A., Khan M.R., Surip S. (2021). Numerical desirability function for adsorption of methylene blue dye by sulfonated pomegranate peel biochar: Modeling, kinetic, isotherm, thermodynamic, and mechanism study. Korean J. Chem. Eng..

[4] Gayathiri M., Pulingam T., Lee K., Sudesh K. (2022). Activated carbon from biomass waste precursors: Factors affecting production and adsorption mechanism. Chemosphere.

[5] Rashid R.A., Mohd Azlan M.I., Kasim M.H. (2018). Adsorptive removal of methylene blue by commercial coconut shell sctivated carbon. Sci. Lett..

[6] Abuelnoor N., AlHajaj A., Khaleel M., Vega L.F., Abu-Zahra M.R. (2021). Activated carbons from biomass-based sources for CO_2_ capture applications. Chemosphere.

[7] Foo K.Y., Hameed B.H. (2012). Factors affecting the carbon yield and adsorption capability of the mangosteen peel activated carbon prepared by microwave assisted K_2_CO_3_ activation. Chem. Eng. J..

[8] Nasrullah A., Saad B., Bhat A., Khan A.S., Danish M., Isa M.H., Naeem A. (2019). Mangosteen peel waste as a sustainable precursor for high surface area mesoporous activated carbon: Characterization and application for methylene blue removal. J. Clean. Prod..

[9] Alacabey İ. (2022). Antibiotic Removal from the Aquatic Environment with Activated Carbon Produced from Pumpkin Seeds. Molecules.

[10] Zubrik A., Matik M., Hredzák S., Lovás M., Danková Z., Kováčová M., Briančin J. (2017). Preparation of chemically activated carbon from waste biomass by single-stage and two-stage pyrolysis. J. Clean Prod..

[11] Zheng S., Zhang J., Deng H., Du Y., Shi X. (2021). Chitin derived nitrogen-doped porous carbons with ultrahigh specific surface area and tailored hierarchical porosity for high performance supercapacitors. J. Bioresour. Bioprod..

[12] Zheng J., Yan B., Feng L., Zhang Q., Zhang C., Yang W., He S. (2022). Potassium citrate assisted synthesis of hierarchical porous carbon materials for high performance supercapacitors. Diam. Relat. Mater..

[13] El-Nemr M.A., El Nemr A., Hassaan M.A., Ragab S., Tedone L., De Mastro G., Pantaleo A. (2022). Microporous Activated Carbon from *Pisum sativum* Pods Using Various Activation Methods and Tested for Adsorption of Acid Orange 7 Dye from Water. Molecules.

[14] Iradukunda Y., Wang G., Li X., Shi G., Hu Y., Luo F., Yi K., Albashir A.I.M., Niu X., Wu Z. (2021). High performance of activated carbons prepared from mangosteen (*Garcinia mangostana*) peels using the hydrothermal process. J. Energy Storage.

[15] Chen S., Jiang S., Jiang H. (2020). A review on conversion of crayfish-shell derivatives to functional materials and their environmental applications. J. Bioresour. Bioprod..

[16] Pezoti O., Cazetta A.L., Souza I.P., Bedin K.C., Martins A.C., Silva T.L., Almeida V.C. (2014). Adsorption studies of methylene blue onto ZnCl_2_-activated carbon produced from buriti shells (*Mauritia flexuosa* L.). J. Ind. Eng. Chem..

[17] Luo X., Cai Y., Liu L., Zeng J. (2019). Cr (VI) adsorption performance and mechanism of an effective activated carbon prepared from bagasse with a one-step pyrolysis and ZnCl_2_ activation method. Cellulose.

[18] Zheng N.-Y., Lee M., Lin Y.-L., Samannan B. (2020). Microwave-assisted wet co-torrefaction of food sludge and lignocellulose biowaste for biochar production and nutrient recovery. Process. Saf. Environ. Prot..

[19] Razzak S.A., Farooque M.O., Alsheikh Z., Alsheikhmohamad L., Alkuroud D., Alfayez A., Hossain S.Z., Hossain M.M. (2022). A comprehensive review on conventional and biological-driven heavy metals removal from industrial wastewater. Environ. Adv..

[20] Shabir M., Yasin M., Hussain M., Shafiq I., Akhter P., Nizami A.-S., Jeon B.-H., Park Y.-K. (2022). A review on recent advances in the treatment of dye-polluted wastewater. J. Ind. Eng. Chem..

[21] Iqbal Z., Tanweer M.S., Alam M. (2022). Recent advances in adsorptive removal of wastewater pollutants by chemically modified metal oxides: A review. J. Water Process. Eng..

[22] Dao M.U., Le H.S., Hoang H.Y., Tran V.A., Doan V.D., Le T.T.N., Sirotkin A. (2021). Natural core-shell structure activated carbon beads derived from *Litsea glutinosa* seeds for removal of methylene blue: Facile preparation, characterization, and adsorption properties. Environ. Res..

[23] Padmini M., Balaganapathi T., Thilakan P. (2022). Rutile-TiO_2_: Post heat treatment and its influence on the photocatalytic degradation of MB dye. Ceram. Int..

[24] Lu C., Yang J., Khan A., Yang J., Li Q., Wang G. (2022). A highly efficient technique to simultaneously remove acidic and basic dyes using magnetic ion-exchange microbeads. J. Environ. Manag..

[25] Bencheqroun Z., Sahin N.E., Soares O.S., Pereira M.F., Zaitan H., Nawdali M., Rombi E., Fonseca A.M., Parpot P., Neves I.C. (2022). Fe (III)-exchanged zeolites as efficient electrocatalysts for Fenton-like oxidation of dyes in aqueous phase. J. Environ. Chem. Eng..

[26] Jawad A.H., Abdulhameed A.S., Wilson L.D., Hanafiah M.A.K.M., Nawawi W.I., ALOthman Z.A., Rizwan Khan M. (2021). Fabrication of schiff’s base chitosan-glutaraldehyde/activated charcoal composite for cationic dye removal: Optimization using response surface methodology. J. Polym. Environ..

[27] Subrahmanya T.M., Widakdo J., Mani S., Austria H.F.M., Hung W.S., Makari H.K., Lai J.Y. (2022). An eco-friendly and reusable syringe filter membrane for the efficient removal of dyes from water via low pressure filtration assisted self-assembling of graphene oxide and SBA-15/PDA. J. Clean Prod..

[28] Khan SAHussain D., Abbasi N., Khan T.A. (2022). Deciphering the adsorption potential of a functionalized green hydrogel nanocomposite for aspartame from aqueous phase. Chemosphere.

[29] Dalvand A., Nabizadeh R., Ganjali M.R., Khoobi M., Nazmara S., Mahvi A.H. (2016). Modeling of Reactive Blue 19 azo dye removal from colored textile wastewater using L-arginine-functionalized Fe_3_O_4_ nanoparticles: Optimization, reusability, kinetic and equilibrium studies. J. Magn. Magn. Mater..

[30] Sing K.S. (1985). Reporting physisorption data for gas/solid systems with special reference to the determination of surface area and porosity (Recommendations 1984). Pure Appl. Chem..

[31] Qi L., Tang X., Wang Z., Peng X. (2017). Pore characterization of different types of coal from coal and gas outburst disaster sites using low temperature nitrogen adsorption approach. Int. J. Min. Sci. Technol..

[32] Hu L., Peng Y., Wu F., Peng S., Li J., Liu Z. (2017). Tubular activated carbons made from cotton stalk for dynamic adsorption of airborne toluene. J. Taiwan Inst. Chem. Eng..

[33] Nasrullah A., Khan A.S., Bhat A., Din I.U., Inayat A., Muhammad N., Bakhsh E.M., Khan S.B. (2021). Effect of short time ball milling on physicochemical and adsorption performance of activated carbon prepared from mangosteen peel waste. Renew. Energy.

[34] Hadi S., Taheri E., Amin M.M., Fatehizadeh A., Lima E.C. (2021). Fabrication of activated carbon from pomegranate husk by dual consecutive chemical activation for 4-chlorophenol adsorption. Environ. Sci. Pollut. Res..

[35] Kongsune P., Rattanapan S., Chanajaree R. (2021). The removal of Pb^2+^ from aqueous solution using mangosteen peel activated carbon: Isotherm, kinetic, thermodynamic and binding energy calculation. Groundw. Sustain. Dev..

[36] Kutluay S., Temel F. (2021). Silica gel based new adsorbent having enhanced VOC dynamic adsorption/desorption performance. Colloids Surf. A Physicochem. Eng. Asp..

[37] Abdulhameed A.S., Jawad A.H., Ridwan M., Khadiran T., Wilson L.D., Yaseen Z.M. (2022). Chitosan/Carbon-Doped TiO_2_ Composite for Adsorption of Two Anionic Dyes in Solution and Gaseous SO2 Capture: Experimental Modeling and Optimization. J. Polym. Environ..

[38] Tang Y., Lin T., Jiang C., Zhao Y., Ai S. (2021). Renewable adsorbents from carboxylate-modified agro-forestry residues for efficient removal of methylene blue dye. J. Phys. Chem. Solids.

[39] Shengli S., Junping L., Qi L., Fangru N., Jia F., Shulian X. (2018). Optimized preparation of *Phragmites australis* activated carbon using the Box-Behnken method and desirability function to remove hydroquinone. Ecotoxicol. Environ. Saf..

[40] Lagergren S. (1898). Zur theorie der sogenannten adsorption geloster stoffe. Vet. Akad. Handl..

[41] Ho Y.S., McKay G. (1998). Sorption of dye from aqueous solution by peat. Chem. Eng. J..

[42] Jawad A.H., Abdulhameed A.S., Surip S.N., Sabar S. (2020). Adsorptive performance of carbon modified chitosan biopolymer for cationic dye removal: Kinetic, isotherm, thermodynamic, and mechanism study. Int. J. Environ. Anal. Chem..

[43] Khan S.A., Abbasi N., Hussain D., Khan T.A. (2022). Sustainable Mitigation of Paracetamol with a Novel Dual-Functionalized Pullulan/Kaolin Hydrogel Nanocomposite from Simulated Wastewater. Langmuir.

[44] Freundlich H.M.F. (1906). Over the adsorption in solution. J. Phys. Chem..

[45] Temkin M.I. (1940). Kinetics of ammonia synthesis on promoted iron catalysts. Acta Physiochim. URSS.

[46] Langmuir I. (1918). The adsorption of gases on plane surfaces of glass, mica and platinum. J. Am. Chem. Soc..

[47] Hoseinzadeh H., Hayati B., Ghaheh F.S., Seifpanahi-Shabani K., Mahmoodi N.M. (2021). Development of room temperature synthesized and functionalized metal-organic framework/graphene oxide composite and pollutant adsorption ability. Mater. Res. Bullet.

[48] El Nemr A., Shoaib A.G., El Sikaily A., Mohamed A.E.-D.A., Hassan A.F. (2021). Evaluation of cationic Methylene Blue dye removal by high surface area mesoporous activated carbon derived from *Ulva lactuca*. Environ. Processes.

[49] Jaramillo-Martínez D., Buitrago-Sierra R., López D. (2022). Use of Palm Oil Waste for Activated Carbons Production and Its Application in Methylene Blue Removal. ChemistrySelect.

[50] Mohd Ramli M.R., Shoparwe N.F., Ahmad M.A. (2022). Methylene Blue Removal Using Activated Carbon Adsorbent from Jengkol Peel: Kinetic and Mass Transfer Studies. Arab. J. Sci. Eng..

[51] Do T.H., Dung N.Q., Chu M.N., Van Kiet D., Ngan T.T.K., Van Tan L. (2021). Study on methylene blue adsorption of activated carbon made from *Moringa oleifera* leaf. Mater. Today Proc..

[52] Baytar O., Ceyhan A.A., Şahin Ö. (2021). Production of activated carbon from *Elaeagnus angustifolia* seeds using H_3_PO_4_ activator and methylene blue and malachite green adsorption. Int. J. Phytoremediat..

[53] Gao M., Xu D., Gao Y., Chen G., Zhai R., Huang X., Xu X., Wang J., Yang X., Liu G. (2021). Mussel-inspired triple bionic adsorbent: Facile preparation of layered double hydroxide@ polydopamine@ metal-polyphenol networks and their selective adsorption of dyes in single and binary systems. J. Hazard. Mater..

[54] Li W., Wei H., Liu Y., Li S., Wang G., Han H. (2021). Fabrication of novel starch-based composite hydrogel microspheres combining Diels-Alder reaction with spray drying for MB adsorption. J. Environ. Chem. Eng..

[55] Jawad A.H., Abdulhameed A.S., Bahrudin N.N., Hum N.N.M.F., Surip S.N., Syed-Hassan S.S.A., Sabar S. (2021). Microporous activated carbon developed from KOH activated biomass waste: Surface mechanistic study of methylene blue dye adsorption. Water Sci. Technol..

[56] Mahapatra U., Chatterjee A., Das C., Manna A.K. (2021). Adsorptive removal of hexavalent chromium and methylene blue from simulated solution by activated carbon synthesized from natural rubber industry biosludge. Environ. Technol. Innov..

